# Association of height loss and cardiovascular disease: Data from a large Korean cohort

**DOI:** 10.3389/fcvm.2022.1026597

**Published:** 2022-11-04

**Authors:** Jeonggeun Moon, Pyung Chun Oh, Kyounghoon Lee, Ho-Jun Jang, Tae-Hoon Kim, Sang-Don Park, Sung Woo Kwon, Min Gyu Kong, Jon Suh, Woong Chol Kang

**Affiliations:** ^1^Division of Cardiology, Department of Internal Medicine, Gil Medical Center, Gachon University College of Medicine, Incheon, South Korea; ^2^Department of Cardiology, Sejong General Hospital, Bucheon, South Korea; ^3^Division of Cardiology, CHA Medical Center, Ilsan Hospital, Ilsan, South Korea; ^4^Department of Cardiology, Inha University Hospital, Incheon, South Korea; ^5^Department of Cardiology, Soonchunhyang University Bucheon Hospital, Bucheon, South Korea

**Keywords:** height loss, CVD, MACCE, aging, cardiovascular disease

## Abstract

**Background:**

Height declines with age, and its degree differs among individuals. Despite epidemiologic evidence for the inverse relationship between adult height and cardiovascular disease (CVD) incidence, the clinical significance of height loss in CVD remains to be elucidated. Therefore, this study investigated the association between height loss and CVD incidence.

**Methods:**

In total, 127,573 Korean participants were enrolled; their heights were monitored from 2002 to 2011. The annual height loss (cm/year) was the difference between the first and last height measurements within the observation period divided by the number of years. The participants were classified as Group 1 (height loss: <0.3 cm/year; *n* = 102,554), Group 2 (height loss: 0.3– < 0.6 cm/year; *n* = 17,324), or Group 3 (height loss: ≥0.6 cm/year; *n* = 7,695).

**Results:**

The cumulative major adverse cardiac and cerebral event (MACCE: cardiac death, non-fatal myocardial infarction, and unplanned hospitalization for heart failure or stroke) incidence rate was 3.6% for Group 1, 4.5% for Group 2, and 5.2% for Group 3. Group 2 (hazard ratio [HR] = 1.27, 95% confidence interval [CI] = 1.17–1.37) and Group 3 (HR = 1.46, 95% CI = 1.32–1.62) had a significantly higher incidence of MACCE than Group 1. In the model adjusted for age, sex, comorbidities, income level, body mass index, smoking, and drinking status, the MACCE risk was higher in Group 2 (HR = 1.11, 95% CI = 1.07–1.20) and Group 3 (HR = 1.25, 95% CI = 1.13–1.39) than in Group 1.

**Conclusion:**

The degree of height loss was independently associated with CVD occurrences in the Korean population.

## Introduction

Growing evidence suggests that short height is associated with cardiovascular disease (CVD) occurrence (1–4), although a rationale for this epidemiological finding remains unclear. Thus, genetically determined height may be a non-modifiable indicator for increased CVD risk. The maximal stature of a given individual is determined in the late teen years (5). However, height declines with aging due to senile changes in the musculoskeletal system (6). The degree of height loss varies among individuals based on the severity of osteoporosis or sarcopenia, which are significant contributors to stature decrease and have a known association with mortality (7–9). Hence, it can be hypothesized that the degree of height loss is correlated with future CVD occurrence. This topic was investigated once in elderly British men two decades ago, and the authors observed that marked height loss (≥3 cm over the preceding two decades) was an independent CVD risk factor (10). However, data from different countries, eras, and ethnicity are needed to confirm this hypothesis because anthropometric parameters, such as height, are influenced by demographic characteristics and the socioeconomic status of the society. The Republic of Korea is suitable for the research for that purpose because it is homogenous as regards ethnicity, and there is a government-driven annual medical check-up/treatment data for all eligible Koreans. This study used a large Korean cohort to investigate the relationship between height loss and CVD prevalence.

## Materials and methods

### Study samples

The National Health Insurance Service (NHIS) of the Republic of Korea operates a mandatory public insurance program for all citizens and supports public health policy and research activities by developing and maintaining the National Health Information Database (11). This study was performed using NHIS data.

**FIGURE 1:**
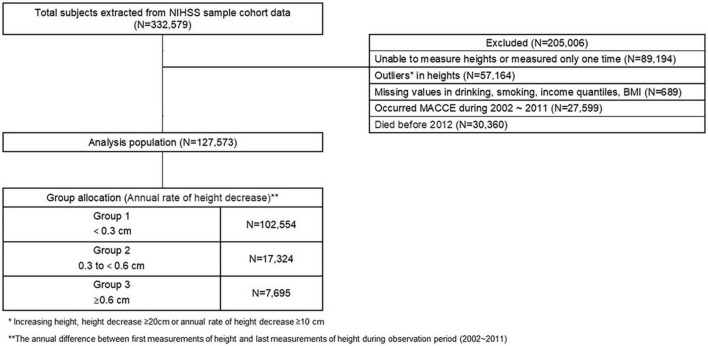
A flow diagram of the study population.

Eligible participants were ethnically Korean, ≥40 years, underwent medical examinations in the Republic of Korea between January 1, 2002, and December 31, 2015; 332,579 individuals were eligible. The exclusion was based on the following: 89,194 individuals had only one height measurement, 57,164 had unreliable height data (such as increasing height, a decrease of ≥20 cm, or an annual decrease rate of ≥10 cm/year), 689 had missing demographic data during the observation period (2002–2011), 27,599 had major adverse cardiac and cerebral events (MACCE) during the observation period, and 30,360 died before 2012, which was the beginning of the outcome monitoring period. As a result, 127,573 individuals were included in the analysis (Figure 1).

### Height and height loss measurements

Height was measured to the nearest 0.1 cm using a stadiometer with the participants standing upright. The observation period for measuring the decrease in height (measured in cm) was ten years (2002–2011). Each participant was included in the study at the time of the first height measurement recording. Heights were measured annually, and the interval between each height measurement was a year or more. The annual height decrease rate (cm/year) was calculated as the difference between the first and the last height measurement within the observation period divided by the number of years. According to annual height decrease rate, participants were classified as Group 1 (reference; height loss: <0.3 cm/year; *n* = 102,554), Group 2 (height loss: 0.3– < 0.6 cm/year; *n* = 17,324), or Group 3 (height loss: ≥0.6 cm/year; *n* = 7,695; Figure 1).

### Outcome definitions and monitoring

Using the Korean Standard Classification of Diseases (KCD-7), which is based on the International Statistical Classification of Diseases and Related Health Problems, MACCE included cardiac death (acute myocardial infarction [MI] [I21], heart failure [I50, I130, I132, I110], cardiac arrest [I46], all with death), non-fatal MI, unplanned hospitalization for heart failure (HF), and stroke (10). Stroke included ischemic and hemorrhagic pathologies. The demographic variables of sex, age, income deciles (1st to 10th deciles), diabetes mellitus, dyslipidemia, hypertension, smoking (individuals who answered “I still smoke” to their smoking status were considered smokers), drinking (individuals who drank > two times per week were considered drinkers), and body mass index (BMI, kg/m^2^) were collected. The BMI was calculated using body weight measured to the nearest 0.1 kg at enrolment. For stratified analysis, those ≥ 60 years were considered elderly (prone to height loss). Women ≥ 50 years were arbitrarily defined as “menopausal” (after which height prominently decreases) based on the previously reported mean menopausal age of Korean women (12).

The primary endpoint was MACCE occurrences between 2012 and 2015. Participants whose last height measurement was at least five years after the first measurement between 2002 and 2011 were included in this study. The incidence of MACCE was collectively followed-up between 2012 and 2015. The start date of MACCE incidences from the last height measurement differed for each participant; therefore, those who developed MACCE before 2012 were excluded from the data set.

### Statistical analyses

Differences in demographic characteristics based on the annual height decrease rate were evaluated using the Chi-square test and the Kruskal–Wallis test. MACCE hazard ratios (HR) and 95% confidence intervals (CI) were calculated based on the annual height decrease rate using a Cox proportional-hazard model adjusted for sex, age, income deciles, diabetes, dyslipidemia, hypertension, smoking, drinking, and BMI. The proportional assumption of the Cox analysis was conducted for Cox proportional-hazard modeling. In univariate analysis, a Log-rank test was conducted to select significant variables. Subsequently, multiple analyses were conducted using Cox’s proportional hazard model. Factors affecting height decreases were analyzed using multivariate linear regression and Kaplan–Meier survival curves and presented to estimate the cumulative MACCE incidence rates with time. Data presents the stratified analyses for the elderly and menopausal populations. All the analyses were performed using the SAS 9.4 software (SAS Institute, Cary, NC, USA) at a statistical significance level of α = 0.05.

## Results

### Study population and baseline characteristics

The overall number of height measurements was 4.1 ± 2.0 (4.3 ± 2.0 times for Group 1, 3.5 ± 1.6 times for Group 2, and 2.8 ± 1.3 times for Group 3). The overall time gap between the first and the last height measurements was 69 ± 28 months (median: 72, interquartile range [IQR, 47–94]) and 73 ± 27 months (median: 78, IQR [51–96]) for Group 1, 58 ± 27 months (median: 57, IQR [32–77]) for Group 2, and 39 ± 27 months (median: 30, IQR [19–55]) for Group 3. Table 1 presents the demographic and clinical characteristic distributions based on the height decrease rate. There were 57,623 males (45.2%) and 69,950 females (54.8%). Group 1 had the highest proportion of males, and Group 3 had the highest proportion of females with a significant inter-group difference (*p* < 0.0001). The highest number of participants were in their 50 s, while the lowest were in their 90 s, with a significant inter-group difference (*p* < 0.0001). The highest number of participants was in the 10th decile of income, and the lowest number was in the 5th decile; this inter-group difference was also significant (*p* < 0.0001). Overall, 48, 488 participants (38.0%) had hypertension, and Group 2 had the highest proportion of patients with hypertension; the inter-group difference was significant (*p* < 0.0001). Further, 44,619 participants (35.0%) had dyslipidemia, and Group 1 had the largest number; the inter-group difference was also significant (*p* < 0.0001). In total, 20,004 participants (15.7%) were smokers, and Group 1 had the highest proportion; the inter-group difference was significant (*p* = 0.0485). Finally, Group 1 had the highest proportion of drinkers, and the inter-group difference was significant (*p* < 0.0001).

**TABLE 1:** Study population baseline characteristics.

	Total	Annual rate of height decrease^†^			*p*-value^††^
		Group 1	Group 2	Group 3	
		
	(*N* = 127,573)	(*N* = 102,554)	(*N* = 17,324)	(*N* = 7,695)	
		
	*n* (%)	*n* (%)	*n* (%)	*n* (%)	
Sex					<0.0001
Male	57,623 (45.2)	48,053 (46.9)	6,780 (39.1)	2,790 (36.3)	
Female	69,950 (54.8)	54,501 (53.1)	10,544 (60.9)	4,905 (63.7)	
Age					<0.0001
40–49 years	17,826 (14.0)	13,359 (13.0)	2,835 (16.4)	1,632 (21.2)	
50–59 years	55,529 (43.5)	47,056 (45.9)	6,121 (35.3)	2,352 (30.6)	
60–69 years	34,497 (27.0)	27,863 (27.2)	4,725 (27.3)	1,909 (24.8)	
70–79 years	17,434 (13.7)	12,826 (12.5)	3,129 (18.1)	1,479 (19.2)	
80–89 years	2,264 (1.8)	1,436 (1.4)	507 (2.9)	321 (4.2)	
≥90 years	23 (0.0)	14 (0.0)	7 (0.0)	2 (0.0)	
Income deciles					<0.0001
Decile 1	11,399 (8.9)	8,870 (8.7)	1,669 (9.6)	860 (11.2)	
Decile 2	9,722 (7.6)	7,619 (7.4)	1,437 (8.3)	666 (8.7)	
Decile 3	9,616 (7.5)	7,609 (7.4)	1,346 (7.8)	661 (8.6)	
Decile 4	9,372 (7.4)	7,375 (7.2)	1,306 (7.5)	691 (9.0)	
Decile 5	9,365 (7.3)	7,488 (7.3)	1,303 (7.5)	574 (7.5)	
Decile 6	11,135 (8.7)	8,950 (8.7)	1,506 (8.7)	679 (8.8)	
Decile 7	12,455 (9.8)	9,980 (9.7)	1,734 (10.0)	741 (9.6)	
Decile 8	14,853 (11.6)	12,006 (11.7)	2,019 (11.7)	828 (10.8)	
Decile 9	18,168 (14.2)	14,803 (14.4)	2,396 (13.8)	969 (12.6)	
Decile 10	21,488 (16.8)	17,854 (17.4)	2,608 (15.1)	1,026 (13.3)	
Hypertension					
Yes	48,488 (38.0)	38,632 (37.7)	6,828 (39.4)	3,028 (39.4)	<0.0001
No	79,085 (62.0)	63,922 (62.3)	10,496 (60.6)	4,667 (60.7)	
Diabetes mellitus					0.0769
Yes	29,468 (23.1)	23,554 (23.0)	4,101 (23.7)	1,813 (23.6)	
No	98,105 (76.9)	79,000 (77.0)	13,223 (76.3)	5,882 (76.4)	
Dyslipidemia					<0.0001
Yes	44,619 (35.0)	36,179 (35.3)	5,949 (34.3)	2,491 (32.4)	
No	82,954 (65.0)	66,375 (64.7)	11,375 (65.7)	5,204 (67.6)	
Smoke					0.0485
Yes	20,004 (15.7)	16,205 (15.8)	2,617 (15.1)	1,182 (15.4)	
No	107,569 (84.3)	86,349 (84.2)	14,707 (84.9)	6,513 (84.6)	
Drink					<0.0001
Yes	47,178 (37.0)	38,978 (38.0)	5,776 (33.3)	2,424 (31.5)	
No	80,395 (63.0)	63,576 (62.0)	11,548 (66.7)	5,271 (68.5)	
Baseline height (cm)					<0.0001
Mean	161.27	161.47	160.44	160.43	Group 1–Group 2^s^,
Standard deviation	8.39	8.37	8.35	8.59	Group 1–Group 3^s^
Body mass index (kg/m^2^)					0.0144
Mean	23.97	23.95	24.04	24.01	Group 1–Group 2^s^
Standard deviation	2.98	2.95	3.09	3.19	

^†^Annual rate of height decrease: Group 1 (<0.3 cm), Group 2 (0.3 to <0.6 cm), and Group 3 (≥0.6 cm).

^††^Chi-square test and the Kruskal–Wallis test.

If there were missing values in sex, age, and income deciles, we imputed them using the closest ones from 2012. We regarded it as a Yes if there was at least one disease.

In case of smoke, drink, and body mass index, the values in 2012 were the criteria. If there were missing values in those variables, we imputed them by the values in 2011.

^s^Significant by *post hoc* test.

### Outcomes and height loss associations

Table 2 presents the cumulative MACCE incidence based on the ranges of the 4-year height decrease. The cumulative MACCE incidence rates were 3.6% for Group 1, 4.5% for Group 2, and 5.2% for Group 3. The rates were 4.3% in Group 1, 5.0% in Group 2, and 6.1% in Group 3 among men, and 3.0% in Group 1, 4.2% in Group 2, and 4.7% in Group 3 among women.

**TABLE 2:** The cumulative adverse cardiovascular and cerebral event incidence rates per the annual height decrease rate.

	All			Male			Female		
	
	Annual rate of height decrease^††^			Annual rate of height decrease^††^			Annual rate of height decrease^††^		
	
	Group 1	Group 2	Group 3	Group 1	Group 2	Group 3	Group 1	Group 2	Group 3
			
	(*N* = 102,554)	(*N* = 17,324)	(*N* = 7,695)	(*N* = 48,053)	(*N* = 6,780)	(*N* = 2,790)	(*N* = 54,501)	(*N* = 10,544)	(*N* = 4,905)
			
Cumulative incidence rate^†^	*n* (%)	*n* (%)	*n* (%)	*n* (%)	*n* (%)	*n* (%)	*n* (%)	*n* (%)	*n* (%)
**MACCE**									
1st year	892 (0.9)	205 (1.2)	95 (1.2)	501 (1.0)	97 (1.4)	42 (1.5)	391 (0.7)	108 (1.0)	53 (1.1)
2nd year	1,817 (1.8)	391 (2.3)	183 (2.4)	1,006 (2.1)	176 (2.6)	78 (2.8)	811 (1.5)	215 (2.0)	105 (2.1)
3rd year	2,696 (2.6)	580 (3.4)	295 (3.8)	1,473 (3.1)	255 (3.8)	124 (4.4)	1,223 (2.2)	325 (3.1)	171 (3.5)
4th year	3,687 (3.6)	785 (4.5)	401 (5.2)	2,042 (4.3)	338 (5.0)	169 (6.1)	1,645 (3.0)	447 (4.2)	232 (4.7)
All	9,092 (8.9)	1,961 (11.3)	974 (12.7)	5,022 (10.5)	866 (12.8)	413 (14.8)	4,070 (7.5)	1,095 (10.4)	561 (11.4)
**Cardiac death**									
1st year	24 (0.0)	5 (0.0)	6 (0.1)	18 (0.0)	3 (0.0)	3 (0.1)	6 (0.0)	2 (0.0)	3 (0.1)
2nd year	85 (0.1)	12 (0.1)	9 (0.1)	58 (0.1)	7 (0.1)	6 (0.2)	27 (0.1)	5 (0.1)	3 (0.1)
3rd year	149 (0.2)	29 (0.2)	23 (0.3)	94 (0.2)	16 (0.2)	11 (0.4)	55 (0.1)	13 (0.1)	12 (0.2)
4th year	245 (0.2)	50 (0.3)	42 (0.6)	155 (0.3)	27 (0.4)	19 (0.7)	90 (0.2)	23 (0.2)	23 (0.5)
All	503 (0.5)	96 (0.6)	80 (1.1)	325 (0.7)	53 (0.8)	39 (1.4)	178 (0.3)	43 (0.4)	41 (0.8)
**Non-fatal MI**									
1st year	114 (0.1)	31 (0.2)	10 (0.1)	84 (0.2)	20 (0.3)	6 (0.2)	30 (0.1)	11 (0.1)	4 (0.1)
2nd year	232 (0.2)	52 (0.3)	21 (0.3)	159 (0.3)	30 (0.4)	12 (0.4)	73 (0.1)	22 (0.2)	9 (0.2)
3rd year	365 (0.4)	75 (0.4)	36 (0.5)	252 (0.5)	44 (0.7)	22 (0.8)	113 (0.2)	31 (0.3)	14 (0.3)
4th year	504 (0.5)	100 (0.6)	51 (0.7)	351 (0.7)	55 (0.8)	31 (1.1)	153 (0.3)	45 (0.4)	20 (0.4)
All	1,215 (1.2)	258 (1.5)	118 (1.5)	846 (1.8)	149 (2.2)	71 (2.6)	369 (0.7)	109 (1.0)	47 (1.0)
**Stroke**									
1st year	737 (0.7)	171 (1.0)	72 (0.9)	394 (0.8)	79 (1.2)	30 (1.1)	343 (0.6)	92 (0.9)	42 (0.9)
2nd year	1,472 (1.4)	323 (1.9)	149 (1.9)	781 (1.6)	140 (2.1)	60 (2.2)	691 (1.3)	183 (1.7)	89 (1.8)
3rd year	2,154 (2.1)	464 (2.7)	227 (3.0)	1,122 (2.3)	193 (2.9)	89 (3.2)	1,032 (1.9)	271 (2.6)	138 (2.8)
4th year	2,876 (2.8)	620 (3.6)	296 (3.9)	1,520 (3.2)	255 (3.8)	116 (4.2)	1,356 (2.5)	365 (3.5)	180 (3.7)
All	7,239 (7.1)	1,578 (9.1)	744 (9.7)	3,817 (7.9)	667 (9.8)	295 (10.6)	3,422 (6.3)	911 (8.6)	449 (9.2)
**Hospitalization for HF**									
1st year	56 (0.1)	11 (0.1)	8 (0.1)	35 (0.1)	6 (0.1)	3 (0.1)	21 (0.0)	5 (0.1)	5 (0.1)
2nd year	116 (0.1)	27 (0.2)	14 (0.2)	69 (0.1)	17 (0.3)	6 (0.2)	47 (0.1)	10 (0.1)	8 (0.2)
3rd year	177 (0.2)	47 (0.3)	29 (0.4)	103 (0.2)	24 (0.4)	11 (0.4)	74 (0.1)	23 (0.2)	18 (0.4)
4th year	270 (0.3)	65 (0.4)	42 (0.6)	152 (0.3)	28 (0.4)	17 (0.6)	118 (0.2)	37 (0.4)	25 (0.5)
All	619 (0.6)	150 (0.9)	93 (1.2)	359 (0.7)	75 (1.1)	37 (1.3)	260 (0.5)	75 (0.7)	56 (1.1)

MACCE, major adverse cardiovascular and cerebral events; MI, myocardial infarction; HF, heart failure.

^†^After observing the degrees of height reduction for 10 years from 2002, we calculated the cumulative incidence rate of MACCE from January 2012 annually.

^††^Annual rate of height decrease: Group 1 (<0.3 cm), Group 2 (0.3 to <0.6 cm), and Group 3 (≥0.6 cm).

**FIGURE 2:**
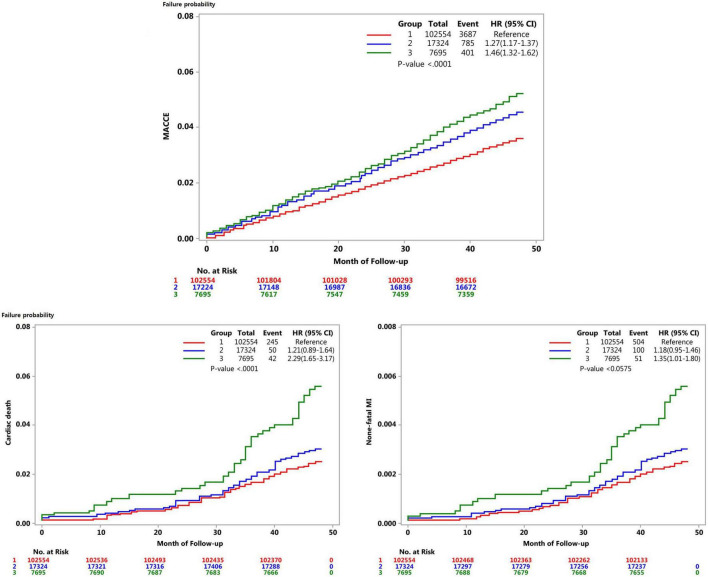
Kaplan–Meier curves for major adverse cardiovascular and cerebral events.

Figure 2 presents the Kaplan–Meier survival curves for the MACCE incidence rates based on the extent of height decrease. The cumulative MACCE incidence rate was highest in Group 3 (HR = 1.46, 95% CI = 1.32–1.62), followed by Group 2 (HR = 1.27, 95% CI = 1.17–1.37) and Group 1 (Reference); the inter-group difference was significant (*p* < 0.0001). All the MACCE components, including cardiac death (HR = 2.29, 95% CI = 1.65–3.17), non-fatal MI (HR = 1.35, 95% CI = 1.01–1.80), and stroke (HR = 1.38, 95% CI = 1.22–1.55) occurred more frequently in Group 3 than in Group 1.

Table 3 presents the Cox proportional-hazard model results for the annual height decrease rate and MACCE incidences. In the crude model, the MACCE and cardiac death risks were 1.46 (95% CI = 1.32–1.62) and 2.29 (95% CI = 1.65–3.17) times higher in Group 3 than in Group 1, respectively, and the inter-group difference was significant, considering the 95% CI. In the adjusted model, the risks of MACCE (HR = 1.25, 95% CI = 1.13–1.39), cardiac death (HR = 1.67, 95% CI = 1.20–2.33), stroke (HR = 1.18, 95% CI = 1.04–1.33), and hospitalization for HF (HR = 1.56, 95% CI = 1.12–2.17) were higher in Group 3 than in Group 1; the inter-group difference was significant. The subgroup analysis of the elderly population (≥60 years) demonstrated similar results (Tables 4, 5). In females, height loss increased dramatically after menopause. The analysis of premenopausal versus menopausal groups demonstrated that MACCE rarely occurred in premenopausal women (Tables 6, 7), and height loss was significantly associated with MACCE in postmenopausal women (≥50 years).

**TABLE 3:** Hazard ratios of adverse cardiovascular and cerebral events per the annual height decrease rate.

	Annual rate of height decrease^††^				
	Group 1	Group 2		Group 3	
Cardiovascular disease	(ref.)	HR	95% CI	HR	95% CI
**Crude model**					
MACCE	1	1.27	1.17, 1.37	1.46	1.32, 1.62
Cardiac death	1	1.21	0.89, 1.64	2.29	1.65, 3.17
Non-fatal MI	1	1.18	0.95, 1.46	1.35	1.01, 1.80
Stroke	1	1.28	1.18, 1.40	1.38	1.22, 1.56
Hospitalization for HF	1	1.43	1.09, 1.87	2.08	1.50, 2.87
**Adjusted model^**†**^**					
MACCE	1	1.11	1.07, 1.20	1.25	1.13, 1.39
Cardiac death	1	0.96	0.71, 1.30	1.67	1.20, 2.33
Non-fatal MI	1	1.12	0.90, 1.39	1.30	0.97, 1.73
Stroke	1	1.12	1.02, 1.22	1.18	1.04, 1.33
Hospitalization for HF	1	1.15	0.88, 1.51	1.56	1.12, 2.17

MACCE, major adverse cardiovascular and cerebral events; MI, myocardial infarction; HF, heart failure.

^†^Sex, age, income deciles, diabetes, dyslipidemia, hypertension, smoke, alcohol use, and body mass index were adjusted.

^††^Annual rate of height decrease: Group 1 (<0.3 cm), Group 2 (0.3 to <0.6 cm), and Group 3 (≥0.6 cm).

Cox proportional hazard model.

**TABLE 4:** The cumulative incidence rates of major adverse cardiovascular and cerebral events per the annual height decrease rate in patients aged ≥ 60 years.

	All			Male			Female		
	
	Annual rate of height decrease^††^			Annual rate of height decrease^††^			Annual rate of height decrease^††^		
	
	Group 1	Group 2	Group 3	Group 1	Group 2	Group 3	Group 1	Group 2	Group 3
			
	(*N* = 42,139)	(*N* = 8,368)	(*N* = 3,711)	(*N* = 20,341)	(*N* = 3,365)	(*N* = 1,353)	(*N* = 21,798)	(*N* = 5,003)	(*N* = 2,358)
			
Cumulative incidence rate^†^	*n* (%)	*n* (%)	*n* (%)	*n* (%)	*n* (%)	*n* (%)	*n* (%)	*n* (%)	*n* (%)
**MACCE**									
1st year	637 (1.5)	165 (2.0)	83 (2.2)	355 (1.8)	75 (2.2)	32 (2.4)	282 (1.3)	90 (1.8)	51 (2.2)
2nd year	1,295 (3.1)	306 (3.7)	148 (4.0)	706 (3.5)	135 (4.0)	58 (4.3)	589 (2.7)	171 (3.4)	90 (3.8)
3rd year	1,923 (4.6)	457 (5.5)	244 (6.6)	1,033 (5.1)	194 (5.8)	98 (7.2)	890 (4.1)	263 (5.3)	146 (6.2)
4th year	2,646 (6.3)	620 (7.4)	329 (8.9)	1,439 (7.1)	258 (7.7)	133 (9.8)	1,207 (5.5)	362 (7.2)	196 (8.3)
All	6,501 (15.4)	1,548 (18.5)	804 (21.7)	3,533 (17.5)	662 (19.7)	321 (23.7)	2,968 (13.6)	886 (17.7)	483 (20.5)
**Cardiac death**									
1st year	22 (0.1)	5 (0.1)	6 (0.2)	16 (0.1)	3 (0.1)	3 (0.2)	6 (0.03)	2 (0.04)	3 (0.1)
2nd year	69 (0.2)	11 (0.1)	8 (0.2)	47 (0.2)	6 (0.2)	5 (0.4)	22 (0.1)	5 (0.1)	3 (0.1)
3rd year	122 (0.3)	24 (0.3)	21 (0.6)	77 (0.4)	11 (0.3)	10 (0.7)	45 (0.2)	13 (0.3)	11 (0.5)
4th year	201 (0.5)	43 (0.5)	38 (1.0)	123 (0.6)	20 (0.6)	16 (1.2)	78 (0.4)	23 (0.5)	22 (0.9)
All	414 (1.0)	83 (1.0)	73 (2.0)	263 (1.3)	40 (1.2)	34 (2.5)	151 (0.7)	43 (0.9)	39 (1.6)
**Non-fatal MI**									
1st year	77 (0.2)	25 (0.3)	8 (0.2)	55 (0.3)	16 (0.5)	4 (0.3)	22 (0.1)	9 (0.2)	4 (0.2)
2nd year	148 (0.4)	41 (0.5)	15 (0.4)	95 (0.5)	24 (0.7)	6 (0.4)	53 (0.2)	17 (0.3)	9 (0.4)
3rd year	232 (0.6)	58 (0.7)	29 (0.8)	145 (0.7)	33 (1.0)	15 (1.1)	87 (0.4)	25 (0.5)	14 (0.6)
4th year	318 (0.8)	77 (0.9)	38 (1.0)	207 (1.0)	40 (1.2)	18 (1.3)	111 (0.5)	37 (0.7)	20 (0.9)
All	775 (1.8)	201 (2.4)	90 (2.4)	502 (2.5)	113 (3.4)	43 (3.1)	273 (1.2)	88 (1.7)	47 (2.1)
**Stroke**									
1st year	529 (1.3)	139 (1.7)	62 (1.7)	284 (1.4)	62 (1.8)	22 (1.6)	245 (1.1)	77 (1.5)	40 (1.7)
2nd year	1,063 (2.5)	253 (3.0)	121 (3.3)	568 (2.8)	107 (3.2)	46 (3.4)	495 (2.3)	146 (2.9)	75 (3.2)
3rd year	1,559 (3.7)	367 (4.4)	187 (5.0)	822 (4.0)	149 (4.4)	70 (5.2)	737 (3.4)	218 (4.4)	117 (5.0)
4th year	2,091 (5.0)	487 (5.8)	243 (6.6)	1,112 (5.5)	197 (5.9)	94 (7.0)	979 (4.5)	290 (5.8)	149 (6.3)
All	5,242 (12.4)	1,246 (14.9)	613 (16.5)	2,786 (13.7)	515 (15.3)	232 (17.2)	2,456 (11.3)	731 (14.6)	381 (16.2)
**Hospitalization for HF**									
1st year	44 (0.1)	8 (0.1)	8 (0.2)	26 (0.1)	4 (0.1)	3 (0.2)	18 (0.1)	4 (0.1)	5 (0.2)
2nd year	90 (0.2)	22 (0.3)	13 (0.4)	48 (0.2)	14 (0.4)	6 (0.4)	42 (0.2)	8 (0.2)	7 (0.3)
3rd year	135 (0.3)	41 (0.5)	25 (0.7)	71 (0.4)	21 (0.6)	11 (0.8)	64 (0.3)	20 (0.4)	14 (0.6)
4th year	208 (0.5)	59 (0.7)	37 (1.0)	107 (0.5)	25 (0.7)	17 (1.3)	101 (0.5)	34 (0.7)	20 (0.9)
All	477 (1.1)	130 (1.6)	83 (2.2)	252 (1.2)	64 (1.8)	37 (2.7)	225 (1.1)	66 (1.4)	46 (2.0)

MACCE, major adverse cardiovascular and cerebral events; MI, myocardial infarction; HF, heart failure.

^†^After observing the degrees of height reduction for 10 years from 2002, we annually calculated cumulative incidence rate of MACCE from January of 2012.

^††^Annual rate of height decrease: Group 1 (<0.3 cm), Group 2 (0.3–<0.6 cm), and Group 3 (≥0.6 cm).

**TABLE 5:** The hazard ratios of major adverse cardiovascular and cerebral events per the annual height decrease rate in patients aged ≥ 60 years.

	Annual rate of height decrease^††^				
	Group 1	Group 2		Group 3	
Cardiovascular disease	(ref.)	HR	95% CI	HR	95% CI
**Crude model**					
**MACCE**	1	1.19	1.09, 1.30	1.43	1.28, 1.67
Cardiac death	1	1.08	0.78, 1.50	2.15	1.52, 3.04
Non-fatal MI	1	1.22	0.95, 1.57	1.36	0.97, 1.90
Stroke	1	1.18	1.07, 1.30	1.33	1.17, 1.52
Hospitalization for HF	1	1.43	1.07, 1.91	2.03	1.43, 2.87
**Adjusted model^**†**^**					
**MACCE**	1	1.10	1.00, 1.20	1.26	1.12, 1.41
Cardiac death	1	0.97	0.67, 1.30	1.71	1.20, 2.43
Non-fatal MI	1	1.20	0.93, 1.54	1.30	0.92, 1.82
Stroke	1	1.09	0.99, 1.20	1.18	1.03, 1.35
Hospitalization for HF	1	1.22	0.91, 1.63	1.56	1.09, 2.22

MACCE, major adverse cardiovascular and cerebral events; MI, myocardial infarction; HF, heart failure.

^†^Sex, age, income deciles, diabetes, dyslipidemia, osteoporosis, hypertension, smoke, drink, BMI were adjusted.

^††^Annual rate of height decrease: Group 1 (<0.3 cm), Group 2 (0.3 to <0.6 cm), and Group 3 (≥0.6 cm).

Cox proportional hazard model.

**TABLE 6:** The cumulative incidence rates of major adverse cardiovascular and cerebral events per the annual height decrease rate in female patients based on menopausal status.

	No			Yes		
	
	Annual rate of height decrease^††^			Annual rate of height decrease^††^		
	
	Group 1	Group 2	Group 3	Group 1	Group 2	Group 3
		
	(*N* = 9,070)	(*N* = 1,939)	(*N* = 1,098)	(*N* = 45,431)	(*N* = 8,605)	(*N* = 3,807)
		
Cumulative incidence rate^†^	*n* (%)	*n* (%)	*n* (%)	*n* (%)	*n* (%)	*n* (%)
**MACCE**						
1st year	14 (0.2)	0 (0.0)	0 (0.0)	377 (0.8)	108 (1.3)	53 (1.4)
2nd year	31 (0.3)	1 (0.1)	2 (0.2)	780 (1.7)	214 (2.5)	103 (2.7)
3rd year	50 (0.6)	4 (0.2)	6 (0.6)	1,173 (2.6)	321 (3.7)	165 (4.3)
4th year	68 (0.8)	7 (0.4)	7 (0.6)	1,577 (3.5)	440 (5.1)	225 (5.9)
All	163 (1.8)	12 (0.6)	15 (1.4)	3,907 (8.6)	1,083 (12.6)	546 (14.3)
**Cardiac death**						
1st year	0 (0.0)	0 (0.0)	0 (0.0)	6 (0.0)	2 (0.0)	3 (0.1)
2nd year	2 (0.0)	0 (0.0)	0 (0.0)	25 (0.1)	5 (0.1)	3 (0.1)
3rd year	6 (0.1)	0 (0.0)	1 (0.1)	49 (0.1)	13 (0.2)	11 (0.3)
4th year	7 (0.1)	0 (0.0)	1 (0.1)	83 (0.2)	23 (0.3)	22 (0.6)
All	15 (0.2)	0 (0.0)	2 (0.2)	163 (0.4)	43 (0.5)	39 (1.0)
**Non-fatal MI**						
1st year	1 (0.0)	0 (0.0)	0 (0.0)	29 (0.1)	11 (0.1)	4 (0.1)
2nd year	3 (0.0)	0 (0.0)	0 (0.0)	70 (0.2)	22 (0.3)	9 (0.2)
3rd year	4 (0.0)	0 (0.0)	0 (0.0)	109 (0.2)	31 (0.4)	14 (0.4)
4th year	6 (0.1)	0 (0.0)	0 (0.0)	147 (0.3)	45 (0.5)	20 (0.5)
All	14 (0.2)	0 (0.0)	0 (0.0)	355 (0.8)	109 (1.3)	47 (1.3)
**Stroke**						
1st year	13 (0.1)	0 (0.0)	0 (0.0)	330 (0.7)	92 (1.1)	42 (1.1)
2nd year	26 (0.3)	1 (0.1)	1 (0.1)	665 (1.5)	182 (2.1)	88 (2.3)
3rd year	41 (0.5)	3 (0.2)	3 (0.3)	991 (2.2)	268 (3.1)	135 (3.6)
4th year	56 (0.6)	6 (0.3)	4 (0.4)	1,300 (2.9)	359 (4.2)	176 (4.6)
All	136 (1.5)	10 (0.5)	8 (0.7)	3,286 (7.2)	901 (10.5)	441 (11.6)
**Hospitalization for HF**						
1st year	0 (0.0)	0 (0.0)	0 (0.0)	21 (0.1)	5 (0.1)	5 (0.1)
2nd year	0 (0.0)	0 (0.0)	1 (0.1)	47 (0.1)	10 (0.1)	7 (0.2)
3rd year	1 (0.0)	1 (0.1)	3 (0.3)	73 (0.2)	22 (0.3)	15 (0.4)
4th year	1 (0.0)	1 (0.1)	3 (0.3)	117 (0.3)	36 (0.4)	22 (0.6)
All	2 (0.0)	2 (0.1)	7 (0.6)	258 (0.6)	73 (0.9)	49 (1.3)

MACCE, major adverse cardiovascular and cerebral events; MI, myocardial infarction; HF, heart failure.

^†^After observing the degrees of height reduction for 10 years from 2002, we annually calculated cumulative incidence rate of MACCE from January of 2012.

^††^Annual rate of height decrease: Group 1 (<0.3 cm), Group 2 (0.3 to <0.6 cm), and Group 3 (≥0.6 cm).

**TABLE 7:** The hazard ratios of major adverse cardiovascular and cerebral events per the annual height decrease rate in female patients based on menopausal status.

	No					Yes				
	Annual rate of height decrease^††^					Annual rate of height decrease^††^				
	Group 1	Group 2		Group 3		Group 1	Group 2		Group 3	
Cardiovascular disease	(ref.)	HR	95% CI	HR	95% CI	(ref.)	HR	95% CI	HR	95% CI
**Crude model**										
**MACCE**	1	0.48	0.22, 1.05	0.85	0.39, 1.85	1	1.49	1.34, 1.65	1.72	1.50, 1.98
Cardiac death	1	0.00	–	1.18	0.15, 9.59	1	1.46	0.92, 2.32	3.17	1.98, 5.07
Non-fatal MI	1	0.00	–	0.00	–	1	1.62	1.16, 2.26	1.63	1.02, 2.59
Stroke	1	0.50	0.22, 1.16	0.59	0.21, 1.62	1	1.47	1.31, 1.65	1.63	1.39, 1.91
Hospitalization for HF	1	4.68	0.29, 74.77	24.88	2.58, 238.48	1	1.63	1.12, 2.36	2.25	1.43, 3.54
**Adjusted model^**†**^**										
**MACCE**	1	0.47	0.22, 1.03	0.83	0.38, 1.82	1	1.22	1.09, 1.35	1.27	1.11, 1.47
Cardiac death	1	0.00	–	1.13	0.14, 9.22	1	1.00	0.63, 1.59	1.74	1.08, 2.81
Non-fatal MI	1	0.00	–	0.00	–	1	1.33	0.95, 1.87	1.20	0.74, 1.92
Stroke	1	0.49	0.21, 1.14	0.57	0.21, 1.58	1	1.22	1.09, 1.37	1.24	1.06, 1.46
Hospitalization for HF	1	4.57	0.29, 73.28	23.69	2.45, 229.50	1	1.15	0.79, 1.67	1.30	0.82, 2.07

MACCE, major adverse cardiovascular and cerebral events; MI, myocardial infarction; HF, heart failure.

^†^Age, income deciles, diabetes, dyslipidemia, osteoporosis, hypertension, smoke, drink, body mass index were adjusted.

^††^Annual rate of height decrease: Group 1 (<0.3 cm), Group 2 (0.3 to <0.6 cm), and Group 3 (≥0.6 cm).

Cox proportional hazard model.

### Height loss determinants

Table 8 presents the multivariate linear regression results on the demographic and clinical characteristics and the annual height decrease. Significant variables affecting the decrease in height included sex, age, low-income, hypertension, dyslipidemia, smoking, drinking, BMI, and baseline height (*p* < 0.0001). Diabetes did not affect the annual height decrease. Particularly, sex (female), age ≥ 60 years, low-income, hypertension, smoking and baseline height positively affect the decrease in height.

**TABLE 8:** Factors associated with affecting the annual height decrease.

	Estimate	Standard error	*T*-value	*P*-value
Intercept	–0.284	0.029	–9.91	<0.0001
Sex (female)	0.072	0.003	23.73	<0.0001
Age (>60)	0.051	0.002	25.11	<0.0001
Low-income	0.021	0.002	10.99	<0.0001
Hypertension	0.007	0.002	3.60	0.0003
Diabetes	0.002	0.002	0.98	0.3267
Dyslipidemia	–0.017	0.002	–8.31	<0.0001
Smoking	0.025	0.003	8.86	<0.0001
Drinking	–0.008	0.002	–3.51	0.0005
BMI (<25)	–0.011	0.002	–5.91	<0.0001
Baseline height	0.003	0.0002	15.02	<0.0001

*R*^2^ = 0.0111, Adj *R*^2^ = 0.0110, *F*-value = 142.93, *P*-value ≤0.0001.

Multivariate linear regression.

## Discussion

In this current research using Korean big data, we observed that the degree of height loss was independently associated with an increased risk of MACCE in a dose-dependent manner. Subgroup analyses of the elderly and postmenopausal women, who have a greater decline in height, also demonstrated this association. These results parallel the British males’ data (*n* = 4,213) published approximately two decades ago and supported the hypothesis that loss of height is more than a common aging process and is a marker for increased CVD risk (10). Compared to that study, the foremost strength of this study is the large sample size. In addition, our study included approximately 54.8% of female participants that were not included in the British data. Homogeneity in the study sample is also noteworthy, as all data were obtained from one country in a relatively short time frame, and the cohort was composed of one ethnicity: Korean. Adult height and its loss are influenced by many factors. As regards confounders affecting height and its loss, the study sample was relatively free from the effects of ethnicity and the chronological change in national socioeconomic status. Based on our study results, patients with significant height decreases should be monitored for CVD occurrence in clinical practice because it might represent an increased risk of CVD. Additionally, considering that determinants of height loss included older age, female sex, hypertension, smoking, baseline height, and low-income level, more attention should be paid to such individuals. The prevention and treatment of senile changes in the musculoskeletal system could also be helpful for cardiovascular health.

For decades, the inverse relationship between adult height and CVD has been repeatedly reported in epidemiological investigations. However, no single theory could explain the mechanism for the phenomenon. Although we and our other colleagues have claimed that dyslipidemia, arterial stiffening represented by a higher pulse wave velocity, or diastolic dysfunction of the heart serve as links between short height and CVD occurrences and outcomes (13–17), entirely satisfactory explanations are still lacking. In this study, we revealed that height loss is associated with increased CVD risk, and we reckon this is clinically meaningful because (1) An individual’s maximum height is genetically determined and is a non-modifiable factor; (2) Conversely, loss of height resulting from senile bone, muscle, and joint changes, is affected by environmental or personal factors, and thus, might be modifiable. The pathophysiology of osteoporosis and sarcopenia, which are responsible for senile height loss, is poorly understood and likely multifactorial; nonetheless, some factors can be targeted for intervention, such as physical inactivity, alcohol use, tobacco use, vitamin D deficiency, or an unhealthy diet. Osteoporosis, characterized by reduced bone density and increased fracture risk, is the primary contributor to height loss by reducing vertebral body strength and inducing the loss of mineral content and trabecular connectivity. Notably, a decrease in stature from osteoporosis and fracture is often remarkable and is likely to reach several centimeters (10, 18). CVD and osteoporosis, which often coexist, are public health challenges with multiple epidemiological links, pathophysiological mechanisms, and economic consequences (19–22). Studies have demonstrated that cardiovascular morbidity and mortality are associated with bone fractures and reduced bone mineral density which is related to vascular calcification, a well-known cardiovascular risk factor (19–22). Additionally, sarcopenia and poor muscle strength, as part of aging, are associated with bone loss and diseased bone structure, eventually resulting in height loss. Osteoporosis and sarcopenia are predictors of mortality and are, at least theoretically, preventable and treatable conditions through environmental modifications and medical interventions (9).

The mechanism relating to the loss of height and subsequent CVD remains unclear. However, the effect of height decrease, a predictor of vertebral fracture, on the respiratory and gastrointestinal systems is noteworthy. Vertebral fractures induce thoracic deformity or pain and disturb pulmonary function subsequently. Conversely, percutaneous vertebroplasty and kyphoplasty for vertebral fractures improve lung function. Further, lung capacity and gastrointestinal function decrease as height declines, potentially leading to less exercise, effort intolerance, early satiety, and poor nutritional status, resulting in sarcopenia and senile fragility (23–28). Weight loss and leanness, attributed to the loss of skeletal muscle mass in the elderly, are risk factors for CVD (the so-called “obesity paradox”) (29–32). Moreover, a musculoskeletal deformity significantly affects psychological conditions, such as depression and anxiety and the quality of life. From a different perspective, however, bone loss and poor bone quality (important determinants of height decline) share common pathophysiological mechanisms with CVD, such as oxidative stress, inflammation, abnormal homocysteine levels, and metabolic risk factors (such as hypertension, diabetes, and dyslipidemia) (33). CVD prevalence increases with age, and the same is true for bone loss and muscle mass decrease: these phenomena might result from shared mechanisms. Hence, from that viewpoint, loss of height might merely be a co-finding or a bystander with CVD rather than a primary cause. Further study is warranted to shed light on the pathophysiological link between loss of height and CVD.

### Limitations

The current investigation was performed based on KCD-7. Thus, biomarkers known as cardiovascular risk factors, such as laboratory findings, were unavailable for analysis. Further, each patient’s medication history was unavailable. The main contributor to height loss is osteoporosis; however, we could not objectively assess each patient’s bone mass density beyond the KCD-7 code. Hence, further study is warranted to clarify the potential pathophysiological association between senile changes in the musculoskeletal system and MACCE. In addition, unreliable height data were excluded from the analysis, and the real change in height cannot be fully distinguished from possible measurement errors. This limitation was due to the retrospective nature of this study. Annual height decrease rate is the main parameter in analysis; however, it could be too crude to represent senile changes in the musculoskeletal system meticulously. In addition, no consideration was given to the potential for non-linear height reduction in data analysis. This was another limitation of this study because it is plausible that serious adverse health events or a period of poor nutrition could lead to a significant loss of height.

## Conclusion

The degree of height loss was independently associated with the occurrence of MACCE in a Korean population, although it has yet to be determined if there is a causal relationship between the two.

## Data availability statement

The data analyzed in this study is subject to the following licenses/restrictions: Restrictions apply to the availability of the raw data, which were used under a policy of the NHIS. The data and materials other than the raw data underlying the study are available from the corresponding author, WCK, on reasonable request. Requests to access these datasets should be directed to WCK, kangwch@gilhospital.com.

## Ethics statement

The studies involving human participants were reviewed and approved by Gachon University Gil Medical Center (IRB No. GFIRB2019-304). The patients/participants provided their written informed consent to participate in this study.

## Author contributions

JGM and WCK designed the study and prepared the manuscript as submitted. JGM analyzed and interpreted the data. WCK supervised the project. All authors reviewed and critically revised the manuscript, read and approved the final manuscript.

## References

[1] Silventoinen K Zdravkovic S Skytthe A McCarron P Herskind AM Koskenvuo M Association between height and coronary heart disease mortality: a prospective study of 35,000 twin pairs. *Am J Epidemiol.* (2006) 163:615–21. 10.1093/aje/kwj081 16484449

[2] Nelson CP Hamby SE Saleheen D Hopewell JC Zeng L Assimes TL Genetically determined height and coronary artery disease. *N Engl J Med.* (2015) 372:1608–18. 10.1056/NEJMoa1404881 25853659 PMC4648271

[3] Park CS Choi EK Han KD Lee HJ Rhee TM Lee SR Association between adult height, myocardial infarction, heart failure, stroke and death: a Korean nationwide population-based study. *Int J Epidemiol.* (2018) 47:289–98. 10.1093/ije/dyx175 29025084

[4] Park YM Moon J Hwang IC Lim H Cho B. Short stature is associated with incident sudden cardiac death in a large Asian cohort. *Heart Rhythm.* (2020) 17:931–6. 10.1016/j.hrthm.2020.01.026 32028046

[5] Solomons NW. Vision of research on human linear growth. *Food Nutr Bull.* (2019) 40:416–31. 10.1177/0379572119885475 31835952

[6] Sorkin JD Muller DC Andres R. Longitudinal change in height of men and women: implications for interpretation of the body mass index: the baltimore longitudinal study of aging. *Am J Epidemiol.* (1999) 150:969–77. 10.1093/oxfordjournals.aje.a010106 10547143

[7] Szulc P Beck TJ Marchand F Delmas PD. Low skeletal muscle mass is associated with poor structural parameters of bone and impaired balance in elderly men–the MINOS study. *J Bone Miner Res.* (2005) 20:721–9. 10.1359/JBMR.041230 15824844

[8] Old JL Calvert M. Vertebral compression fractures in the elderly. *Am Fam Phys.* (2004) 69:111–6. 14727827

[9] Metter EJ Talbot LA Schrager M Conwit R. Skeletal muscle strength as a predictor of all-cause mortality in healthy men. *J Gerontol A Biol Sci Med Sci.* (2002) 57:B359–65. 10.1093/gerona/57.10.b359 12242311

[10] Wannamethee SG Shaper AG Lennon L Whincup PH. Height loss in older men: associations with total mortality and incidence of cardiovascular disease. *Arch Intern Med.* (2006) 166:2546–52. 10.1001/archinte.166.22.2546 17159023

[11] Lee YH Han K Ko SH Ko KS Lee KU. Data analytic process of a nationwide population-based study using national health information database established by national health insurance service. *Diabetes Metab J.* (2016) 40:79–82. 10.4093/dmj.2016.40.1.79 26912157 PMC4768054

[12] Lim HS Kim TH Lee HH Park YH Kim JM Lee BR. Hypertension and age at onset of natural menopause in Korean postmenopausal women: results from the korea national health and nutrition examination survey (2008-2013). *Maturitas.* (2016) 90:17–23. 10.1016/j.maturitas.2016.04.019 27282789

[13] Moon J Lee HJ Kim YJ Kim JY Pak HN Ha JW Short stature and ischemic stroke in nonvalvular atrial fibrillation: new insight into the old observation. *Int J Cardiol.* (2014) 174:541–4. 10.1016/j.ijcard.2014.04.154 24814538

[14] Moon J Suh J Oh PC Lee K Park HW Jang HJ Relation of stature to outcomes in Korean patients undergoing primary percutaneous coronary intervention for acute st-elevation myocardial infarction (from the INTERSTELLAR registry). *Am J Cardiol.* (2016) 118:177–82. 10.1016/j.amjcard.2016.04.046 27236252

[15] Moon J Hwang IC Han SH. Short stature is associated with higher pulse wave velocity in subjects without overt cardiovascular disease. *Medicine.* (2020) 99:e22219. 10.1097/MD.0000000000022219 32991415 PMC7523875

[16] Hwang IC Park YM Kang WC Moon J. Association between height and lipid profile among Korean men: results from the 10-year Korea national health and nutrition examination survey. *Eur J Prev Cardiol.* (2020) 27:2205–7. 10.1177/2047487319877055 31581806

[17] Hwang IC Park YM Kang WC Moon J. Height is associated with dyslipidemia in korean premenopausal women: data from the korea national health and nutrition examination survey. *Cardiology.* (2020) 145:736–9. 10.1159/000509631 32911470

[18] Siminoski K Warshawski RS Jen H Lee K. The accuracy of historical height loss for the detection of vertebral fractures in postmenopausal women. *Osteoporos Int.* (2006) 17:290–6. 10.1007/s00198-005-2017-y 16143833

[19] Farhat GN Cauley JA. The link between osteoporosis and cardiovascular disease. *Clin Cases Miner Bone Metab.* (2008) 5:19–34. 22460842 PMC2781192

[20] Crepaldi G Maggi S. Epidemiologic link between osteoporosis and cardiovascular disease. *J Endocrinol Invest.* (2009) 32:2–5. 19724158

[21] McFarlane SI Muniyappa R Shin JJ Bahtiyar G Sowers JR. Osteoporosis and cardiovascular disease: brittle bones and boned arteries, is there a link. *Endocrine.* (2004) 23:1–10. 10.1385/ENDO:23:1:01 15034190

[22] Whitney C Warburton DE Frohlich J Chan SY McKay H Khan K. Are cardiovascular disease and osteoporosis directly linked. *Sports Med.* (2004) 34:779–807. 10.2165/00007256-200434120-00001 15462612

[23] Lombardi I Oliveira LM Mayer AF Jardim JR Natour J. Evaluation of pulmonary function and quality of life in women with osteoporosis. *Osteoporos Int.* (2005) 16:1247–53. 10.1007/s00198-005-1834-3 15806323

[24] Kjensli A Falch JA Ryg M Blenk T Armbrecht G Diep LM High prevalence of vertebral deformities in COPD patients: relationship to disease severity. *Eur Respir J.* (2009) 33:1018–24. 10.1183/09031936.00073908 19129288

[25] Leech JA Dulberg C Kellie S Pattee L Gay J. Relationship of lung function to severity of osteoporosis in women. *Am Rev Respir Dis.* (1990) 141:68–71. 10.1164/ajrccm/141.1.68 2297189

[26] Ross PD Davis JW Epstein RS Wasnich RD. Pain and disability associated with new vertebral fractures and other spinal conditions. *J Clin Epidemiol.* (1994) 47:231–9. 10.1016/0895-4356(94)90004-3 8138833

[27] Ross PD. Clinical consequences of vertebral fractures. *Am J Med.* (1997) 103:30S–42S; discussion 42S–43S. 10.1016/s0002-9343(97)90025-5. 9302895

[28] Yuan HA Brown CW Phillips FM. Osteoporotic spinal deformity: a biomechanical rationale for the clinical consequences and treatment of vertebral body compression fractures. *J Spinal Disord Tech.* (2004) 17:236–42. 10.1097/00024720-200406000-00012 15167341

[29] Hughes VA Frontera WR Roubenoff R Evans WJ Singh MA. Longitudinal changes in body composition in older men and women: role of body weight change and physical activity. *Am J Clin Nutr.* (2002) 76:473–81. 10.1093/ajcn/76.2.473 12145025

[30] Carnethon MR De Chavez PJ Biggs ML Lewis CE Pankow JS Bertoni AG Association of weight status with mortality in adults with incident diabetes. *JAMA.* (2012) 308:581–90. 10.1001/jama.2012.9282 22871870 PMC3467944

[31] Logue J Walker JJ Leese G Lindsay R McKnight J Morris A Association between BMI measured within a year after diagnosis of type 2 diabetes and mortality. *Diabetes Care.* (2013) 36:887–93. 10.2337/dc12-0944 23139375 PMC3609520

[32] Pischon T Boeing H Hoffmann K Bergmann M Schulze MB Overvad K General and abdominal adiposity and risk of death in Europe. *N Engl J Med.* (2008) 359:2105–20. 10.1056/NEJMoa0801891 19005195

[33] Laroche M Pécourneau V Blain H Breuil V Chapurlat R Cortet B Osteoporosis and ischemic cardiovascular disease. *Joint Bone Spine.* (2017) 84:427–32. 10.1016/j.jbspin.2016.09.022 27838246

